# Botulinum toxin: Bioweapon & magic drug

**Published:** 2010-11

**Authors:** Ram Kumar Dhaked, Manglesh Kumar Singh, Padma Singh, Pallavi Gupta

**Affiliations:** *Biotechnology Division, Defence Research & Development Establishment, Gwalior, India*

**Keywords:** Botulism, botulinum toxin, *Clostridium botulinum*, neurotoxin, proteinase

## Abstract

Botulinum neurotoxins, causative agents of botulism in humans, are produced by *Clostridium botulinum*, an anaerobic spore-former Gram positive bacillus. Botulinum neurotoxin poses a major bioweapon threat because of its extreme potency and lethality; its ease of production, transport, and misuse; and the need for prolonged intensive care among affected persons. A single gram of crystalline toxin, evenly dispersed and inhaled, can kill more than one million people. The basis of the phenomenal potency of botulinum toxin is enzymatic; the toxin is a zinc proteinase that cleaves neuronal vesicle associated proteins responsible for acetylcholine release into the neuromuscular junction. As a military or terrorist weapon, botulinum toxin could be disseminated via aerosol or by contamination of water or food supplies, causing widespread casualties. A fascinating aspect of botulinum toxin research in recent years has been development of the most potent toxin into a molecule of significant therapeutic utility. It is the first biological toxin which is licensed for treatment of human diseases. In the late 1980s, Canada approved use of the toxin to treat strabismus, in 2001 in the removal of facial wrinkles and in 2002, the FDA in the United States followed suit. The present review focuses on both warfare potential and medical uses of botulinum neurotoxin.

## Introduction

*Clostridium botulinum* and botulism, the disease it causes, have been known to man for centuries^1^. Botulism is a severe neuroparalytic disease caused by the action of botulinum neurotoxins (BoNTs) produced by anaerobic spore-forming *C. botulinum* and some of its close relatives^2^. The BoNTs are regarded as the most potent toxins known to mankind^3^. If left untreated, a severe case of botulism leads to death of the patient due to paralysis of respiratory muscles. Although the disease has been known to man since ancient time, Muller in 1870 coined the name ‘botulism’ for the newly described disease^4^. Following the advent of microbiology in the late 19^th^ century, the causative organism was isolated from contaminated meat and recognized as an anaerobic bacillus^5^. Cultivation of the bacillus and its subsequent introduction into animals leading to development of the symptoms of botulism has been reported^6^.

One of the most fascinating aspects in the field of botulinum toxin research in recent years has been application of the most potent toxin in treatment of neurological disorders. It has become the first biological toxin which is licensed as drug for treatment of human diseases. As of January 2008, two BoNT serotypes (A and B) are approved for clinical use in the United States by Food and Drug Administration (FDA). Subsequently, the neurotoxin has become a household name as clients line up at local gyms, parties, and spas for Botox injections, in order to temporarily rid themselves of wrinkles and sweaty armpits. This review provides updated information on warfare potential and medical uses of botulinum neurotoxin.

## Botulism: Disease

All four forms of botulisms (food borne, infant, wound and animal) cause illness through a common pathway regardless of the manner in which the toxin gains systemic access^7^. Botulism initiates with acute weakness of muscles, causing difficulty in speaking and swallowing and double with blurred vision in all forms of diseases. This is followed by a progressive symmetrical flaccid paralysis, descending from the muscles of the head and throat, which in severe cases causes death due to respiratory muscles paralysis^8^. Mental functioning is not impaired by BoNTs, so the patient remains alert and conscious throughout the disease^9^. Botulism is confirmed by detection of BoNT in a patient’s serum or stool, or in a sample of food consumed before onset of illness^10^.

Food-borne botulism is also known as “classical” botulism, as it was the first form of the disease described in literature. Food poisoning due to botulinum toxin emerged as a problem when food preservation became a widespread practice. BoNT is secreted in to food by toxigenic clostridia growing in it under suitable conditions. Ingestion of preformed toxin is responsible for the botulism thus this type of disease represents intoxication rather than an infection, which is the case of other form of human botulisms. In a study of 2622 outbreaks in which BoNT types were determined, 34 per cent were caused by type A, 52 per cent by type B and 12 per cent by type E. Only two food borne outbreaks were assigned to BoNT type F during this period^11^. More than 90 per cent cases of foodborne botulism have been reported due to home prepared or home preserved foods^9^. A wide variety of commercially produced (preserved and non-preserved) foods have caused botulism outbreaks. Examples include foil-wrapped baked potatoes^12^, canned chili sauce^13^, jarred peanuts^14^, packed food^15^, hazelnut yogurt^16^, garlic in oil^17^, carrot juice^18^, and matambre (Argentine meat roll)^19^.

Infant botulism, recognized as a clinical identity over three decades ago^20^, has been the most diagnosed form of botulism in USA since 1979^21^. The initial neurological symptoms of infant botulism are largely the same as in other forms of botulism, but these are usually missed by parents and doctors because the infant can not verbalize them. The case/fatality ratio among hospitalized patients was reported to be less than one per cent^22^. The source of spores for most cases remains unknown, although the most common sources of infection for infants appear to be honey and environmental exposure^23^,^24^. Analysis of infant botulism cases occurring globally from 1996 through 2008 revealed 524 cases in 26 countries representing five continents^25^.

Another form of botulism is analogous to tetanus, in that BoNT is determined from *C. botulinum* growing *in vivo* in abscessed wounds called wound botulism. Most cases occur in physically active young males who are presumable at higher risk of traumatic injuries^22^. Wound botulism has emerged as a small-scale epidemic in San Francisco, USA, among Bay Area drug abusers following subcutaneous injection of heroin^26^. Similarly, in the United Kingdom, bacterial infections (particularly wound botulism) have increased markedly since 2000 among injecting heroin users^27^. Some cases have also been reported in Germany^28^ and in Sweden, where real-time PCR was used to diagnose a case of type E wound botulism^29^. The case / fatality ratio has been rather high (15%)^9^.

Most mammals are susceptible to botulinum neurotoxin and develop botulism with similar clinical features to humans^30^–^32^. A majority of cases are caused by *C. botulinum* group III, although groups I and II are also reported in animal botulism^31^. Horses are very sensitive to BoNTs and equine botulism occurs sporadically worldwide, both as feed poisoning and as toxico-infectious forms^31^. Avian botulism is usually caused by BoNT type C1, to which most birds seem to be susceptible. Botulism is very dangerous in fish farming^33^. Contaminated silage has been reported to cause botulism outbreaks among cattle^34^.

Inhalational botulism is not a natural form of botulism and most likely to be seen on the battlefield, is rare. One incident involving accidental exposure of humans to BoNT/A in a laboratory of Germany was reported in 1961^35^. More data are available on exposure of animals to toxin aerosols. Rhesus monkeys were exposed by inhalation to BoNT/A, in conjunction with toxoid and hyperimmune globulin efficacy trials^36^. Park and Simpson^37^ reported that BoNT/A, an inhalation poison, works by the active process of binding and transcytosis across airway epithelial cells.

Iatrogenic botulism is caused inadvertently by injection of botulinum toxin for therapeutic or cosmetic reasons^38^. Four cases of iatrogenic botulism occurred in December 2004 in Florida following cosmetic injection with a botulinum toxin that was not approved for use in humans^39^.

## Botulism in Indian scenario

Food-borne botulism is thought to be an uncommon clinical condition in India and is rarely reported. First incidence of food borne botulism in India was reported in 1996 involving 34 students with two deaths and toxigenic *C. butyricum* was isolated^40^. Two patients of one family (42 yr old man and his 6 yr old daughter) consumed canned meat products were diagnosed clinically according to CDC guidelines as botulism^41^. Dhaked *et al*^42^ isolated toxigenic clostridia from soil of slaughter house, of which one was confirmed by PCR and mouse protection assay as *C. botulinum* type E. Prevalence and distribution of *C. botulinum* was also studied in fish from coastal and inland areas of India. Types A to D were found to be present on sediments, surface of wild fish and intestine with dominance of *C. botulinum* type C and D^43^,^44^. Recently, multiplex PCR for the detection of *C. botulinum* and *C. perfringens* toxin genes was reported on eight suspected food borne botulism cases^45^.

## *Clostridium botulinum*: Bacterium

Bacteria isolated from the outbreaks of the beginning of the century were not all similar to the Van Ermengem’s strain^46^. The clinical manifestations of the intoxication were all alike, but the cultural characteristics and growth requirement of different isolates differed. By cross neutralization tests of their respective toxins the different *C. botulinum* isolates were divided into two types, A and B^47^. Bacteria were also isolated from animal botulism cases in 1920 and were designated as type C^48^ and D^49^. Thereafter, a serotype E was isolated from fish food^50^. Moller & Scheibel^51^ isolated serotype F and Gimenez & Ciccarelli^52^ serotype G, respectively from a Danish patient and Argentinean soil. Thus, seven distinct serotypes of botulinum toxin have now been isolated, designated A through G. That means one serotype has been isolated approximately every 12 years since Van Ermengem’s original isolation. Serotypes A, B, E and F have been clearly identified in numerous human poisoning episodes. Serotype G has only been identified in a few outbreaks. Serotypes C and D have been found in outbreaks involving various animals. Why humans are typically not poisoned by serotypes C and D is not clear.

Early chromosomal DNA-DNA homology studies^53^ showed that the single species decision did not hold up to modern nucleic acid based taxonomical scrutiny and later *C. botulinum* was divided into three groups I to III^54^. This decision was validated through 16S ribosomal RNA sequence analysis^55^–^57^. The non-disease forming serotype G^52^, found at the time of grouping was termed as *C. botulinum* group IV by Smith & Hobbs^58^, but has subsequently been given a species name of its own, *Clostridium argentinens*^59^. It has been recognized that the botulinum neurotoxins are produced by four distinct groups of *C. botulinum* based on cultural and biochemical properties or DNA-DNA homology. However, in 1986, it was demonstrated that two clostridial species other than *C. botulinum* produced botulinum toxin in three cases of infant botulism, two in Rome, Italy^60^ and one in New Mexico^61^. Type BoNT/E producing *C. butyricum* was isolated from the cases in Rome and BoNT/F producing *C. baratii* from the infant botulism case in New Mexico. Generally a single organism expresses a single toxin type but some strains of *C. botulinum* are also reported to be capable of producing mixtures of two types of toxin, such as A+F, A+B or B+F^2^. In addition, strains that possess unexpressed, ‘silent’ genes have also been reported^62^.

Whole genome sequences of various *C. botulinum* strains along with their plasmids are available in the GenBank depositories. Total 17 *C. botulinum* complete genomes have been sequenced (till Oct, 2009), which include representatives of all the serotypes excepting *C. botulinum* type G^63^–^68^. These genomes provide an excellent opportunity for comparative analysis of *C. botulinum* and will undoubtedly provide valuable insights into the pathogenicity, metabolic diversity and evolution of these organisms.

## Botulinum neurotoxins

Botulinum neurotoxins are the most poisonous poison known to the humankind produced by strains of *C. botulinum*. The lethal dose for a person by the oral route is estimated at 30 ng^69^, by the inhalational route 0.80 to 0.90 µg, and by the intravenous route 0.09 to 0.15 µg^38^. Assuming an average weight of 70 kg each of 5.6 billion people, only 39.2 g of pure BoNT would be sufficient to eradicate humankind^22^. Due to their absolute neurospecificity these neurotoxins do not react with any substrates in the presynaptic motor neurons, BoNTs are extremely toxic^17^. The two most likely mechanisms for use of botulinum toxin as a terrorist weapon include deliberate contamination of food or beverages or via an aerosol release^71^.

In type A, three different sized progenitor toxins with molecular masses of 900 kDa (19 S, LL toxin), 500 kDa (16 S, L toxin) and 300 kDa (12 S, M toxin) were observed^72^–^74^. Types B, C and D strains produce both 16 S and 12 S toxins, whereas types E and F produce 12 S toxins and type G produces only 16 S toxin^75^. Therefore, it was postulated that 19 S and 16 S toxins have both haemagglutinin (HA) and non-toxin non-haemagglutin (NTNH) proteins whereas 12 S toxin is formed by association of NTNH protein only.

The neurotoxin is released as a single polypeptide chain of 150 kDa, which is later nicked to generate two disulphide linked fragments, the heavy chain (H, 100 kDa) and light chain (L, 50 kDa) (Fig.). The H chain is responsible for binding, internalization and membrane translocation, whereas L chain for target modification in the cytosol^76^. The function of L chains has been established as zinc dependent endopeptidases^77^, and the substrates are one of the three proteins of the docking complex responsible for release of acetylcholine from synaptic vesicles. Light chain of types A, C and E acts on SNAP 25^78^–^81^ and VAMP/ synaptobrevin is cleaved by BoNT B, D, F and G along with tetanus neurotoxin^77^,^79^,^82^–^84^ whereas syntaxin is cleaved by BoNT/C^85^,^86^. BoNT is internalized in cholinergic nerve endings and remain in the presynaptic motor neurons causing flaccid paralysis^76^.

**Fig:**
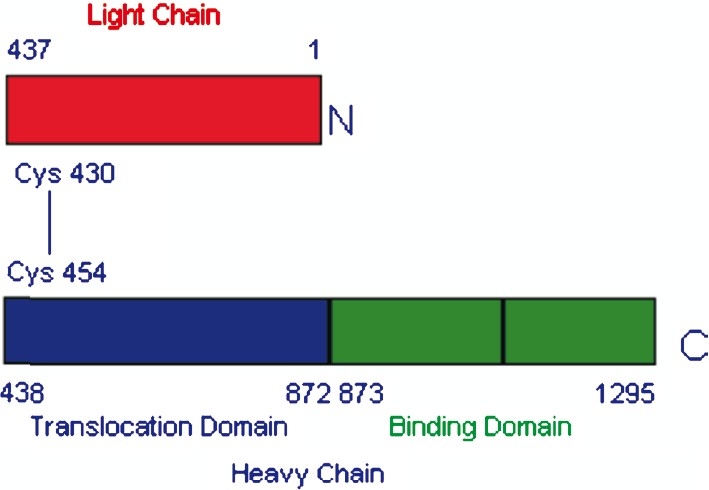
The di-chain structure of a botulinum neurotoxin A (BoNT/A). Botulinum neurotoxins are ~150-kDa proteins, synthesized as single-chain polypeptides and post-translationally nicked to form di-chain molecules. They share the same domain architecture and overall structure. The light and heavy chains of BoNT/A are linked by a single disulphide bond, Cys430–Cys454. The light chain, shown in red, functions as zinc-dependent endopeptidase. The heavy chain comprises two functional domains of roughly equal size. The N-terminal section, shown in blue, is the translocation domain and is thought to be involved in translocation and activation of the LC. The C-terminal section, shown in green, is acting as binding domain.

## Characterization and detection of *Clostridium botulinum* and their toxins

Basic principal of detection and isolation of *C. botulinum* from clinical, food and environmental samples has remained essentially unchanged since E Van Ermengem’s first report, more than a century ago^5^. Isolation of *C. botulinum* almost invariably starts with anaerobic enrichment of the samples in a non selective culture media^87^, *e.g.* Robertson cooked meat medium (CMM) or trypticase-peptone-yeast extract-glucose (TPYG) broth for 3-10 days at 26-35°C. Usually culture are heat (70°C for 10 min) or ethanol treated (50 % for 1 h) prior to plating to get rid off vegetative cells which greatly improves subsequent isolation^88^. Although selective media^89^ for *C. botulinum* have been developed, their use has remained limited. The efficiency of selection in the media has been questioned^90^, since antibiotics used seems to inhibit some strains of type E and to alter the appearance of type G colonies^91^.

The optimal and minimal growth temperatures for group I strains is 35-40 and 10°C, for group II strains 18-25 and 3.3°C, and for group III strains 40 and 15°C, respectively^2^. The cells of all strains of *C. botulinum* are straight to slightly curved sporulating, anaerobic bacilli with round ends, measuring 2 to 20 µM in length and 0.5 to 2 µM in width^31^. The spores are oval and sub-terminal and usually swell to occupy the sporangium^92^. The spores are resistant to heat, desiccation, chemicals, radiation and oxygen which facilitate their survival for very long periods. Most cultures retain Gram stain well, becoming Gram-negative only after sporulation or during late stationary phase^31^.

It is thought that since *C. botulinum* is an anaerobic organism, it will be unable to grow in foods which are exposed to oxygen or in foods which do not have a low oxidation-reduction potential (Eh). Actually, the Eh of the food exposed to oxygen is low enough in most of the food to permit the growth of *C. botulinum*. Even though the maximum growth occurs at an Eh of -350 mV^93^, *C. botulinum* can grow at Eh values as high as + 250 mV^94^. A substantial body of research has shown no growth of *C. botulinum* at pH 4.8 or lower and led to the current government regulation that canned foods at pH 4.6 or lower would be safe without conventional sterilization^95^. One of the first definitive attempts to influence water activity (a_w_) was performed by Denny *et al*^96^ and reported that the growth of *C. botulinum* type A and B was dependent on a_w_ of canned bread and not on moisture content. No toxin was produced in canned breads stored up to two years with a_w_ values ≤ 0.950. Emodi & Lechowich^97^ found that the minimum a_w_ for the growth of type E ranges from 0.972 to 0.978 in a wide variety of solutes. The minimum a_w_ values for the growth of types A and B in food is 0.94 and for type E is 0.97 corresponding to a sodium chloride concentration of 10 and 5 per cent, respectively.

Identity of *C. botulinum* and other BoNT producing clostridia is confirmed by toxin detection. Table Ilists detection limits, field applicability and the types of samples for which BoNT assays were demonstrated.

**Table I:** Performance of existing botulinum toxin assays

Assay	Type of toxin	Time of the assay	Detection limit	Potential for field Diagnostics*	Sample type
Mouse neutralization assay^88^	A, B, C, D, E, F, G	1-4 days	20-30 pg/ml	++	Foods, serum and stool
TRF^98^	A, B	2 h	20-200 pg/ml.	+	Clinical/environmental samples
Fluorometric Biosensor^99^	A, B	uncertain	?	+/-	Aassay buffer and live cells
Modified ELISA^100^	A, B, E, F	6 h	0.6 ng/ml	++	Liquid and solid foods, serum
Micromechanosensor^101^	B	15 min	>8 nM	++	Sample buffer
Mass Spectometry MALDI-TOF-MS/Endopeptidase-MS^102^	A, B, E, F	4 - 16 h	5 pg/ml or lower	+/-	Milk, serum and stool extract
BoNT ALISSA^103^	A	2-3 h	0.5 fg/ml	++	Serum, milk, carrot juice, gelatin and phosphate diluents
Immuno-PCR^104^	A, B, E	4 - 6 h	50 fg/ml	+/-	Carbonate buffer
Liposome PCR assay^105^	A	6 h	0.2 fg/ml	+/-	Carbonate buffer
Enzyme-amplified protein microarray immunoassay^106^	A	10 min	1.4pg/ml	+	Blood and plasma
SPR^170^	B, F	5 min	0.1 pg/ml	+/-	Assay buffer
Ganglioside-liposome immunoassay^108^	A	20 min	15pg/ml	+/-	Assay buffer

* Potential for field diagnostics: ++ high, +intermediate, +/- low

In outbreaks of botulism, it is customary to assay suspected foods, patient’s sera, faeces samples and enrichment cultures for the presence of toxin^91^. Half ml of undiluted toxin preparation with same amount of 1:2, 1:10 and 1:100 diluted antisera in gelatin phosphate buffer should be intra-peritoneally injected in pairs of 15-20 g mice. Through incorporation of serotype testing by Leuchs^46^ and Burke^47^, the method has evolved present day form, the mouse bioassay^88^. The second stage of the mouse lethality test is to identify the serological toxin type by mouse protection assay with specific monovalent (types A-G) antisera. Universally acknowledged for detecting biological activity of BoNTs in samples, the mouse bioassay, although is highly sensitive, has been criticized as being slow, laborious, expensive and lacking in specificity. Furthermore, the increasing public resistance to animal testing makes it clear that there is a need to replace bioassay with reliable *in vitro* test^109^. Recovery of *C. botulinum* from stools or gastric samples with symptoms and signs indicative of botulism is usually sufficient for confirmation. Recovery of the organism from food that does not contain demonstrable toxin is inconclusive. Electrophysiological studies can provide a presumptive diagnosis of botulism in patients with clinical signs of botulism^110^ and can be especially helpful when laboratory tests are negative.

Numerous attempts were made to replace mouse bioassay with immunological based methods i.e., fluorescent antibody test^111^, immunodiffusion^112^, fiber optic biosensor^113^, streptavidin-biotin amplified ELISA^114^ and ELCA (enzyme linked coagulation assay) amplified ELISA^115^. Some of these methods are sensitive enough and used in some laboratories for screening samples, however, any of these methods is so far not authorized for official or clinical use due to their inability to differentiate active and inactive neurotoxin^109^. Recently, PCR-ELISA has been used for the study of prevalence of *C. botulinum* type A, B, E and F in fish and environmental samples in northern France^116^. In another attempt, extreme biological specificity of the BoNTs for proteins VAMP, SNAP25, *etc*., in the nerve cells, has been utilized in a novel ‘second generation’ ELISA, the endopeptidase assay^117^. Attempts are also being made to develop sensitive endopeptidase assay utilizing small fluorigenic peptide substrates for the protease activities of botulinum neurotoxins, serotypes A, B, and F^118^.

PCR-based methods^119^ detecting the botulinum neurotoxin (BoNT) gene was pioneered to replace the time consuming conventional methods and mouse bioassay. Since then different workers^42^,^120^–^125^ have applied this technique for the detection of BoNT genes in epidemics, environmental samples screening and epidemiological prevalence studies. Lindstrom *et al*^126^ have described and detected four BoNT genes namely types A, B, E and F by multiplex PCR.

The first report about genomic characterization of *C. botulinum* was published in 1995 and included MRP analysis of four type A strains by pulsed field gel electrophoresis (PFGE)^127^. Hielm *et al*^128^ described the use of PFGE in genomic analysis of group II C botulinum and found it to be highly discriminating and reproducible. However, not all strains were typeable either due to DNA degradation by active endonucleases or resistance of the cell wall to lysis. The application of rRNA gene restriction pattern analysis (ribotyping) for the genomic characterization of *C. botulinum* groups I and II strains has also been reported^129^. However, the discriminatory power was found to be lower than that of PFGE and there were some difficulties in the interpretation of patterns generated by certain restriction enzymes. Therefore, ribotyping was concluded to be suitable only for taxonomic purposes in *C. botulinum* species identification.

## Protection against botulinum neurotoxin

Since the reported cases of all forms of botulism are rare, vaccination for general population is not warranted on the basis of cost and expected adverse reactions with even the best vaccines. Moreover, vaccination against BoNT will restrict its therapeutic and cosmetic applications in the subjects. There are two basic alternatives for prophylaxis of high risk individuals from botulinum poisoning; active immunization using a vaccine, or passive immunotherapy using immunoglobulin. In cases of wound botulism, the wound should be surgically debrided and antibiotics should be administered (usually penicillin). A pentavalent crude toxoid vaccine (A-E) and a singular F toxoid are investigational drugs distributed by the CDC to military and research workers that might come into contact with toxin^130^. Since these have not acquired FDA approval, these toxoid vaccines are not licensed for general distribution. The impetus to meet FDA requirements is low, because these vaccines require frequent boosters and are toxic due to the formaldehyde used to inactivate the toxins^131^. Efficacy of the pentavalent botulinum toxoid (PBT) was evaluated and antibodies concentrations were found to be significantly higher (≥ 0.25 U) in 99 per cent of the 508 military personnel vaccinated before and after Persian Gulf War^132^.

Even though toxoid vaccines are available, there are numerous shortcomings with their current use^133^. *(i) C. botulinum* being spore former, a dedicated facility is required; *(ii)* yields of toxin production are very low; *(iii)* the toxoiding process involves large quantities of toxin and thus dangerous; *(iv)* toxoid proteins are not purified thus other proteins may influence immunogenicity or reactivity of the vaccine; and *(v)* since the residual levels of formalin are part of the product formulation to prevent reactivation of toxin, the vaccine is reactogenic. The development of a new generation recombinant vaccine could alleviate many of the problems associated with the toxoid. So the alternative approaches to develop vaccines against the botulinum neurotoxins are currently being pursued by several laboratories. Attassi & Oshima^134^ have synthesized a series of overlapping 19 mer peptides that spawned the entire Hc region of BoNT/A and reported as vaccine candidate. Lee *et al*^135^ introduced a gene fragment encoding non-toxic Hc region of BoNT/A into Venezuelan equine encephalitis virus replicon vector to yield high levels of Hc that protected mice against a 10^5^ LD_50_ challenge of BoNT/A. Byrne *et al*^136^ expressed the region of BoNT/A in *Pichia pastoris* and recombinant BoNT/A Hc prevented botulinum intoxication. Immunization of mice with three doses of 1 mg heavy chain of BoNT/B was fully protective when mice were challenged with 10^6^ LD_50_ BoNT/B after 1 year of vaccination^133^. DNA vaccine^137^ fused with signal peptide could protect mice against 10^4^ MLD challenge of BoNT/F. Recently, a single dose of adenovirus-vectored vaccine molecules derived from heavy chain of type C are reported to provide protection against botulism^138^,^139^.

Antitoxin therapy^140^ is more effective if undertaken early in the course of illness. The only antitoxins available are equine antitoxin from CDC (neutralizing antibodies against BoNT/A, /B, and /E) and an investigational heptavalent (against ABCDEFG) antitoxin. BabyBIG®, derived from the blood of human donors vaccinated with a pentavalent (ABCDE) toxoid vaccine, is only available for infant botulism^141^. This is not surprising when one considers that equine antitoxin neutralizes only toxin molecules yet unbound to nerve endings^142^. More than 80 per cent of persons reported with adult botulism in the United States are treated with antitoxin. However, treatment is not without risk, as approximately 9 per cent of persons treated experience hypersensitivity reactions^143^. A human-derived botulism antitoxin, termed “botulism immune globulin”^144^, has been prepared, and a clinical trial of its efficacy when given early in the course of illness is in progress in California.

## Molecular inhibitors against neurotoxin

The BoNT molecule is divided in clear functional domains that can operate independently. This feature provides multiple targets for designing therapeutics to treat botulism. Therapeutics against BoNT can target any of the three steps of mode of action of BoNT: binding, endocytosis/translocation, and endopeptidase activity. Humanized monoclonal antibodies, small peptides, peptide mimetics, receptor mimics, and small molecules targeting active sites are candidates for inhibiting botulinum toxin and may eventually be used in treatment strategies. Studies reported that toosendanin^145^–^147^ (major limonoid constituent of the bark of the tree *M. toosendan*) could protect monkeys from BoNT/A, BoNT/B, and BoNT/E-induced death in a dose dependent fashion when co-administered with, or several hours after, neurotoxin administration. A semisynthetic strategy to identify inhibitors based on toosendanin, has been reported by Fischer *et al*^148^ to protect from BoNT intoxication.

Based on the substrate information, several small peptides have been developed as competitive inhibitors for the BoNT endopeptidase activity. Peptidomimetics and hydroxamic acid-based inhibitors have been developed that display inhibitory effects^149^–^152^ in the high nm range for the light chain of the BoNT serotype A.

Many drug-like small molecule libraries are available commercially as well as in national repositories. Screening these drug-like compounds has become critical in finding new therapeutic candidates. Screening such libraries requires a robust assay feasible for the high throughput screening. Such assays have been developed for screening the endopeptidase activity of BoNT^106^,^107^, making it feasible to find inhibitors against the protease activity of BoNT by screening large library of compounds.

Other target to design antagonists against botulism is to block the binding between BoNTs and their receptors. Cai *et al*^153^ have demonstrated that the quinic acid can inhibit the binding between HcQ and the ganglioside at the concentration of 10 mM. While receptor mimics are valid targets for designing inhibitors against the botulism, like antibody based therapy, the treatment window for such agents is short, since they can only target at the circulation level. Once the toxin gets internalized into the nerve cells, effectiveness of receptor-based inhibitors will be very limited.

Aptamers form unique structures that provide basis for high affinity and specificity towards their targets (proteins or the small molecules). Their specific and tight interactions serve as valuable tools to modulate or block functions of proteins. The screening process for aptamers is popularly termed as SELEX (Systematic Evolution of Ligands through EXponential enrichment). An efficient and easy-to-execute single microbead SELEX approach is developed to generate high affinity ssDNA aptamers against botulinum neurotoxin^154^.

Targeting extracellular neutralization and binding of BoNT to cell surface will provide effective prophylactic treatment and prevention measures to botulism. An effective BoNT-based drug delivery vehicle can be used to directly deliver toxin inhibitors into intoxicated nerve terminal cytosol to reverse the paralysis. Recently, amino dextran based drug delivery vehicle has been reported to deliver BoNT-A antidotes into BoNT-A intoxicated cultured mouse spinal cord cells^155^. This approach can potentially be utilized for targeted drug delivery to treat other neuronal and neuromuscular disorders.

## BoNTs as magic drug

One of the most fascinating aspects on *C. botulinum* in recent years has been development of the most potent toxin into a molecule of significant therapeutic utility. Purified protein derived from the bacterium *C. botulinum* type A was originally developed about three decades ago by US scientists for medical use^156^,^157^. BoNT is the first bacterial toxin licensed by USFDA as ‘occulinum’ a drug for the treatment of blepharospasm in 1989. Botox® (from Allergan), minute amount of purified BoNT/A, is the only botulinum toxin treatment to have undergone the rigorous approval process in 15 countries required to secure a license for the treatment of facial wrinkles. This holds a unique position in that it is a safe and effective medical treatment for a number of highly distressing conditions, while also being used as a cosmetic therapy where there is no underlying medical condition. Lately, BoNT/B^158^ and BoNT/F^159^ were also successfully used to prevent muscles hyperactivity. As of January 2008, two BoNT serotypes (A and B) are approved for clinical use in the United States by Food and Drug Administration (*www.fda.gov*). A carefully purified and defined quantity of the botulinum neurotoxin is injected by a trained surgeon within the spastic muscle which considerably reduce presynaptic outflow of acetylcholine at the neuromuscular junction, with a consequent diminution in muscle hyperactivity/contraction, while leaving some strength for the physiological function. A basal rate of acetylcholine secretion across the synaptic cleft occurs continuously, with each packet of acetylcholine depolarizing the post-synaptic membrane to create miniature end plate potentials (MEPPs). MEPPs summate to maintain the motor end-plate potential (EPP). Botulinum neurotoxins prevent acetylcholine secretion, reducing the frequency and quantity, but not amplitude of MEPPs. The motor EPP is reduced below the muscle membrane threshold and the ability to generate muscle fiber action potentials and subsequent contraction is diminished^160^. These toxins are safe drugs. One reason is that upon injection the protein does not diffuse beyond 2 cm, exerting its paralyzing activity around the injection site with very limited spreading. Several pharmaceutical preparations of botulinum toxins for the treatment of human diseases in ophthalmology, neurology and dermatology are currently marketed under the trade names *Botox*®, *Dysport*® and *Xeomin*® (based on botulinum neurotoxin A), and *Myoblock*® /*Neuroblock*® (based on botulinum neurotoxin B)^161^–^163^. With the exception of Xeomin, which is practically devoid of complexing proteins^164^, the other commercial formulations of botulinum toxins include, besides the neurotoxin, other bacterial complexing haemagglutinins and nonhaemagglutinin proteins as well. Several additional substances (*e.g.*, albumin, sucrose, lactose) are included in these preparations and aim at drug stabilization and facilitation of administration by intramuscular injection. In lyophilized form the toxins may be kept in long storage; however, if diluted with saline for injection, these must be used within a few hours. The biological potency of these preparations is expressed in mouse units. One mouse unit is defined as the intraperitoneally injected quantity of each pharmaceutical product required to kill 50 per cent (LD_50_) of an experimental group of female Swiss-Webster mice, each of 20 g body weight. The US FDA has approved use of these preparations in cervical dystonia, blepharospasm, spasmodic, torticollis, strabismus and glabellar frown lines. These are being used in approximately 150 different indications, *e.g.*, disorders of ocular motility, writer’s cramp, hemi facial spasm and spasticity, achalasia, chronic anal fissure and hyperhidrosis (Table II). The new uses for this ‘wonder drug’ are under constant evaluation, including gastrointestinal smooth muscles and skeletal muscle spasm following CNS injury, cosmetic management of wrinkles^179^ and debarking of dogs^178^. One vial of Botox contains 100 units (U) of purified neurotoxin complex produced by *C. botulinum* type A, 0.5 mg of albumin (human), and 0.9 mg of sodium chloride in a sterile, vacuum-dried form without a preservative. The lethal dose of the Botox preparation for a person of 70 kg is calculated to be 2,500-3,000 units. The recommended dose for large muscles, localized by touch, (*e.g.* gastrocnemius) is 100-400 units, whereas for cosmetic purposes usually less than 30 units are injected directly into the targeted muscle. For smaller muscles or deeper muscles, detected through electrostimulation, (*e.g.* orbicularis oculi) 1-2 sites of injection and a quantity of 3–4 units are effective, whereas a large muscle (*e.g.* gastrocnemius) requires 4-5 injections and 300-400 units^180^,^181^.

**Table II:** Uses of botulinum neurotoxin

Indication	Example
Dystonias^165^	Cervical dystonia, Oromandibular dystonia, Pharyngolaryngial dystonias, Jaw closure/opening dystonias, Occupational cramps, Limb and axial dystonias
Spasticity^166^	Cerebral palsy, Brain injury, Spinal cord injury
Eyelid spasm^167^	Blepharospasm, Hemifacial spasm, Eyelid twitch
Exocrine gland hyperactivity^168^	Focal hyperhidrosis, Relative sialorrohoea, Crocodile tears syndrome,
Movement disorders^169^	Tremors, Bruxism, Tic
Pain syndromes^170^	Migraine, Back spasm
Urinary bladder dysfunction^171^	Sphincter- detrusor dyssenergia, detrusor hyperreflexia,
Opthalmology^172^	Strabismus, Entropion, Protective ptosis
Cosmetology^173^	Hyperactive facial lines-brow lines, Frown lines,
Gastroenterology^174^	Achalasia, Anal fissures, Anismus
Gynecology^175^	Vaginismus
Urology^176^	Sterile prostatitis
Dentistry^177^	Muscle spasm associated with temporomandibular joint pathology
Veterinary^178^	Barking dogs

Inherent in the use of a protein-based therapeutic is the potential for antibody formation leading to a decrease in effectiveness of the treatment. Such secondary non-responders are seen in a relatively low percentage of patients, most commonly requiring large doses of BoNT, often on repeated occasions. Since the majority of the immune response is generated toward the Hc fragment, future protein engineering of hybrid toxins could provide one route to prolong the therapeutic efficacy of BoNT treatment.

## Future directions

Although some progress has been made in recent years, identification and characterization of the protein receptors for the BoNTs and determination of the mechanism of specificity of CNT binding domains for their receptors is an outstanding problem. Further, understanding the mechanism of LC translocation and activation within the motorneuron, including the effects of pH on the tertiary structures of BoNTs, will be crucial for rational design of engineered BoNT therapeutics. Further structural studies on the endopeptidase domains of BoNTs, including the structural basis behind BoNT substrate specificity, might lead to the development of serotype-specific inhibitors.

It has been proposed that the extreme neurospecificity of BoNT heavy chains could be applied to deliver engineered molecule in to nerve cells. This can be achieved by the replacement of light chain with desired therapeutic agent that could be reached in the nerve endings without iatrogenic complications which might otherwise occur^182^. Use of fragments of BoNT for the therapeutics of the future is also exciting. For example, harnessing the properties of the BoNT LC endopeptidase fragments for the creation of a range of ‘designer’ therapeutics is a real possibility following the successful retargeting of the LC/A domain to cells of neuronal and non-neuronal origin^183^. Additionally, the ability of BoNTs to transport large polypeptides across the membranes could be harnessed for the delivery of biopharmaceuticals to cytosolic targets^184^. Derivatives of BoNT/A and BoNT/B can target compounds specifically to human neuroblastoma cells. The therapeutic potential of clostridial toxins is not limited to the neurotoxin for the inhibition of neurotransmitter release, but also has potential as an anticancer drug^62^. The technology termed ‘clostridia directed enzyme pro-drug therapy’ (CDEPT) in which intravenously injected clostridial spores are used to target hypoxic regions of solid tumours. Spores get localized to solid tumours exclusively for germination, as they cannot grow in healthy tissues. Genetic modification of the clostridial host to express anti cancer compounds or pro-drug converting enzymes (as in CEDPT), has the potential to lead the localized destruction of solid tumour tissue.

Botulinum neurotoxins are of great interest to the medical and scientific communities. Despite causing disease, they have become valuable research tools and have wide-ranging applications as pharmaceuticals. As the structure and mechanism of action of the toxins are further dissected, the development of vaccines, serotype-specific inhibitors and novel therapeutics will undoubtedly follow.
